# Patterns of disease occurrence and management, and public health issues among Korean populations based on information and experiences obtained by field epidemiological studies in various situations with episodic stories never been told

**DOI:** 10.4178/epih.2017010

**Published:** 2017-03-16

**Authors:** Hyun-Sul Lim

**Affiliations:** Department of Preventive Medicine, Dongguk University College of Medicine, Gyeongju, Korea


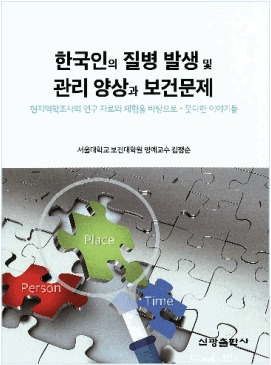


Dr. Joung-Soon Kim, Professor emeritus, Graduate School of Public Health, Seoul National University, is a pioneer of Korean epidemiology and has been awarded the Moran and Dongbaekjang Medals for Civil Merit and the Aquamarine Stripes for Service Merit. At last, a book has been published of her experiences while working as a professor in field epidemiology for over 30 years. How can I express my joy? Here, Professor Kim publishes anecdotes of her experiences in the field that went untold in the academic papers, together with materials relating to the study methods and conclusions derived from her already legendary opus of research. In addition to this, the book is a comprehensive exploration of the meaning of public health science, of new experiences and valuable lessons, and recollections of certain researchers.

The emergence of a disease or abnormal phenomenon now typically results in calls for epidemiological research. Conducting reliable epidemiological studies require experienced researchers; indirect experiences could be useful even when direct experiences are not accessible. However, domestically, there are almost no resources describing methods to perform a successful epidemiological investigation. Given the circumstances, it is no exaggeration to say that this book is the authoritative work in epidemiology.

In Chapter 1 ‘Searching for the causes of diseases of which etiologies are unknown’, Kim describes, like a thriller, the process of discovering the causative pathogens for the first domestic cases of Legionnaires’ disease and leptospirosis, the epidemic of anthrax in Shinan-gun, and lead poisoning in all crew members of the cargo ship.

In Chapter 2 ‘Searching for the causes of epidemics and pathways of transmission in order to prevent further spread of infectious diseases’, Kim gives a vivid account of the processes involved in elucidating pathways of transmission for typhoid fever and cholera epidemics in various peculiar situations.

In Chapter 3 ‘Research on the epidemiological characteristics of persistent life threatening parasitic diseases and on the efficacy of mass treatment among the residents of Jeju-do, an isolated island’, the readers may find a detailed description of methods used to evaluate the effectiveness of projects to eradicate paragonimiasis and filariasis from Jeju-do, learning about disease eradication techniques in the process.

In Chapter 4 ‘Research on the ecological and epidemiological characteristics of *Paragonimus* in Malaysia’, Kim describes her experiences of research in Malaysia, allowing the readers to learn about the requirements of performing research abroad.

In Chapter 5 ‘Epidemiological research to select efficient diagnostic techniques and evaluation criteria for occupational diseases caused by exposure to a harmful environment’, the readers can learn about occupational and environmental epidemiology by looking at epidemiological studies of carbon disulfide poisoning at Wonjin Rayon Co., which resulted in the largest ever population with occupational diseases domestically, and also exhaustive and thorough studies for the defoliants exposure among Korean Vietnam Veterans.

In Chapter 6 ‘Patterns of disease, health management behaviors, and public health issues among Koreans’ and Chapter 7 ‘Mortality and morbidity patterns, and public health issues comparing Koreans in Korea and the ethnic Koreans living in Manchuria’ highlight the essence of descriptive epidemiology, with comparison of diseases in rural and urban residents, and of mortality patterns in the ethnic Koreans living in Manchuria, the Han Chinese, and Koreans; with particular interest in sorting out or differentiate the impacts of causing certain prevalent chronic diseases whether it is related to genetic factors or environmental factors, or both factors mixed.

These writings provide a description on authentic experiences that cannot be found in academic papers. In addition to describing field epidemiological studies that obviously have to be conducted when health is affected by infectious diseases or harmful substances (lead, carbon disulfide, defoliant), this book also presents the stories behind these studies and uses them to demonstrate the meaning of public health science, and shares new experiences and lessons. Kim provides insight on how to recognize the epidemiological hint, how to win the cooperation of experts in the field, how to deal with incompetent and dishonest research organizations, and how to respond to conflicting opinions.

This book is required reading for anyone studying medicine or public health science, especially for those studying epidemiology or conducting epidemiological field surveys. It will also help readers to understand the significance of epidemiology and epidemiological surveys.

Professor Kim is an expert in epidemiology, who has committed to a single field and never hesitated to go wherever the nation needs her. As readers absorb the stories of a true scholar who gave everything to helping others and her country while maintaining the highest ethical standards, they will be able to learn the proper attitude and wisdom of a scholar.

