# Supplementary material for: Patterns of disease occurrence and management, and public health issues among Korean populations based on information and experiences obtained by field epidemiological studies in various situations with episodic stories never been told
Source: Epidemiol Health. 2017 Mar 16;39:e2017010. doi: 10.4178/epih.2017010 (PMC5434220; doi:10.4178/epih.2017010)
Supplement: Supplementary file 1 [file epih-39-e2017010-supplementary.pdf]

Book Review: Patterns of disease occurrence and management, and public health issues among Korean populations based on information and experiences obtained by field epidemiological studies in various situations with episodic stories never been told

Hyun-Sul Lim

Key Message

서평: 한국인의 질병 발생 및 관리 양상과 보건문제 현지역학조사의 연구 자료와 체험을 바탕으로 - 못다한 이야기들

한국 역학의 개척자인 서울대학교 보건대학원 김 정순 명예교수님께서 30여년 동안 교수로 재직하면서 현지역학조사를 수행하면서 경험한 내용을 책으로 출간하셨다. 이미 전설이 된 많은 기념비적인 역학연구를 연구 방법과 결론을 추론해 가는 과정을 자료와 함께 제시하고 학술지를 발표할 때 하지 못한 현지 체험 비화를 수록하였다. 더구나 보건학적 의의와 새로 얻은 경험 및 귀중한 교훈, 일부 연구원의 감회까지 포괄하여 소개하고 있다. 역학이라는 한 분야에 몰두하면서 국민이 필요한 곳에는 어느 곳이나 달려가서 역학 전문가로서 최선을 다하면서 학자의 양심을 끝까지 지킨 참된 학자의 이야기를 읽으면서 학자의 자세와 지혜를 깨닫기 바란다.

서평

임 현술
동국의대 예방의학교실,
경북 경주시 동대로 123 wisewine@dongguk.ac.kr

한국인의 질병 발생 및 관리 양상과 보건문제
현지역학조사의 연구 자료와 체험을 바탕으로 - 못다한 이야기들

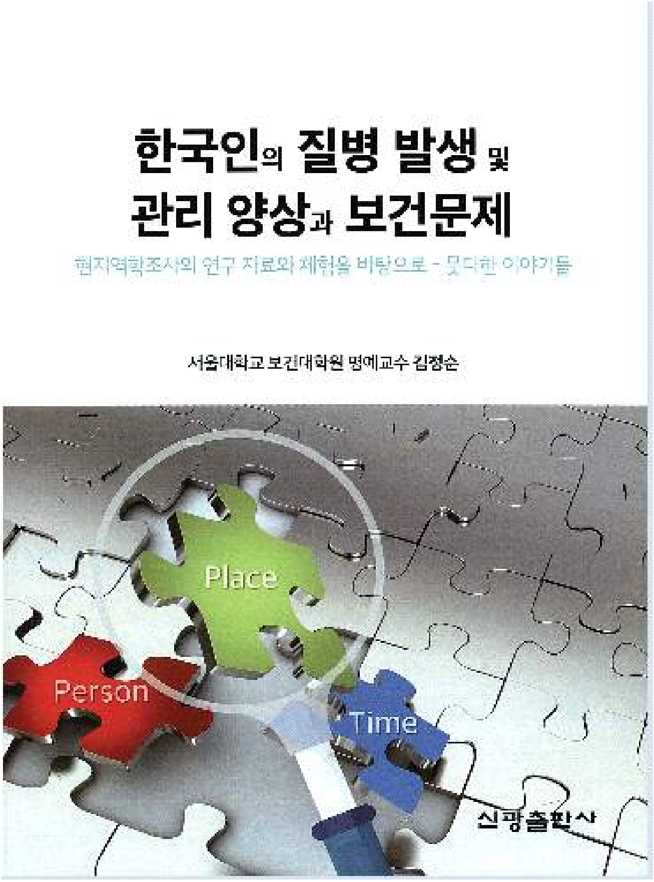

한국 역학의 개척자로 국민훈장 동백장, 옥조근정훈장 및 모란장을 수상한 서울대학교 보건대학원 김 정순 명예교수님께서 30여 년 동안 교수로 재직하면서 현지역학조사를 수행하면서 경험한 내용을 드디어 책으로 출간하셨다. 이 기쁨을 무엇으로 표현할 수 있을까? 이미 전설이 된 많은 기념비적인 역학연구를 연구 방법과 결론을 추론해 가는 과정을 자료와 함께 제시하고 학술지를 발표할 때 하지 못한 현지 체험 비화를 수록하였다. 더구나 보건학적 의의와 새로 얻은 경험 및 귀중한 교훈, 일부 연구원의 감회까지 포괄하여 소개하고

있다.

최근 어떤 질병이나 이상한 현상이 생기면 역학조사를 하여야 한다고 주장한다. 믿을만한 역학조사를 하려면 경험이 중요하며, 경험이 적다면 간접 경험이라도 하여야 할 것이다. 그러나 국내에서는 역학조사를 잘하기 위한 방법을 기술한 자료가 거의 없다. 이러한 때 이 책은 역학조사의 결정판이라고 해도 과언이 아니다.

1장 '원인불명질환(괴질)의 원인을 찾아서'에서는 국내 최초로 밝힌 레지오넬라증 및 렙토스피라증, 또한 신안군에서 발생한 탄저 유행, 외항선원에서 발생한 납 중독에 대하여 원인(균)을 파악해 가는 과정을 추리소설을 보듯이 묘사하고 있다.
2장 '전염병 방역을 위한 유행 원인과 전파경로를 찾아서'에서는 장티푸스 유행들과 콜레라 유행에 대한 전파경로를 밝혀 가는 과정을 박진감 넘치게 기술하고 있다.
3장 '제주도민의 고질적 기생충증의 역학적 특성과 집단 치료의 퇴치효과에 관한 연구'에서는 제주도 폐흡충증과 사상충증의 퇴치사업과 효과를 평가하는 방법을 자세히 기술하여 질병 퇴치 기법을 터득할 수 있다.
4장 '말레이시아 폐흡충의 생태학적, 역학적 특성에 관한 연구'에서는 말레이시아에서 연구 체험을 기술하여 국외에서 연구할 때 고려할 사항을 배울 수 있다.
5장 '유해 환경 노출에 의한 직업병의 판정기준 설정을 위한 효율적 진단방법의 선정을 위한 역학적 연구'에서는 국내 최대의 직업병 환자가 발생한 ㈜원진 레이온의 이황화탄소 중독증 역학조사와 파월 국군장병의 고엽제 역학조사의 진행 과정 등을 통하여 직업 및 환경역학을 접할 수 있다.
6장 '한국인의 상병 양상 및 관리행태와 보건문제'와 7장 '이주 한국인, 만주 조선족의 사망 및 상병양상과 보건문제'를 통하여 농촌지역 주민과 도시 영세지역 주민의 상병조사, 만주 조선족, 한족 그리고 한국인의 사망양상의 비교로 기술역학의 진수를 엿볼 수 있다.

감염병과 유해물질(납, 이황화탄소, 고엽제)에 의한 건강장애가 발생하면 당연히 수행하여야 하는 현지역학조사 뿐만 아니라 역학조사 뒷이야기, 보건학적 의의와 새로 얻은 경험과 교훈을 제시하여 논문을 통하여 획득할 수 없는 경험의 실체에 접할 수 있다. 하늘의 도움을 얻는 방법, 기자의 기지를 인지할

수 있는 방법, 현지 전문가의 협조를 구하는 방법, 무능하고 부정직한 연구기관에 대처하는 방법 및 반대 견해에 대하여 대응하는 방법 등 무궁무진한 지혜를 터득할 수 있다.

이 책은 의학과 보건학, 특히 역학을 공부하거나 현지에서 역학조사를 수행하는 모든 분들에게 참고서로 대용할만한 필독서이다. 일반인에게는 역학 및 역학조사의 의미를 이해하는데 크게 도움이 될 것이다.

역학이라는 한 분야에 몰두하면서 국민이 필요한 곳에는 어느 곳이나 달려가서 역학 전문가로서 최선을 다하면서 학자의 양심을 끝까지 지킨 참된 학자의 이야기를 읽으면서 학자의 자세와 지혜를 깨닫기 바란다.
